# Optimization of Processing Parameters for Extraction of Amylase Enzyme from Dragon (*Hylocereus polyrhizus*) Peel Using Response Surface Methodology

**DOI:** 10.1155/2014/640949

**Published:** 2014-06-23

**Authors:** Mehrnoush Amid, Mohd Yazid Abdul Manap, Norkhanani Zohdi

**Affiliations:** Department of Food Technology, Faculty of Food Science and Technology, Universiti Putra Malaysia (UPM), 43400 Serdang, Selangor, Malaysia

## Abstract

The main goal of this study was to investigate the effect of extraction conditions on the enzymatic properties of thermoacidic amylase enzyme derived from dragon peel. The studied extraction variables were the buffer-to-sample (B/S) ratio (1 : 2 to 1 : 6, w/w), temperature (−18°C to 25°), mixing time (60 to 180 seconds), and the pH of the buffer (2.0 to 8.0). The results indicate that the enzyme extraction conditions exhibited the least significant (*P* < 0.05) effect on temperature stability. Conversely, the extraction conditions had the most significant (*P* < 0.05) effect on the specific activity and pH stability. The results also reveal that the main effect of the B/S ratio, followed by its interaction with the pH of the buffer, was significant (*P* < 0.05) among most of the response variables studied. The optimum extraction condition caused the amylase to achieve high enzyme activity (648.4 U), specific activity (14.2 U/mg), temperature stability (88.4%), pH stability (85.2%), surfactant agent stability (87.2%), and storage stability (90.3%).

## 1. Introduction

Amylases are enzymes that catalyse the initial hydrolysis of starch to shorter oligosaccharides; an important step towards transforming starch into single units [1]. This class of enzyme holds the maximum market share of enzyme sales, with its most extensive application being in the food industry [2]. With the advances in new frontiers in biotechnology, the spectrum of amylase application has also expanded to automatic dishwashing detergents as well as textile desizing and the pulp and paper industry [3]. Amylases with desirable properties, such as high thermostability, low pH stability, raw starch digestibility, and the utilisation of a high concentration of starch, can be very useful in related applications. Amylase is also used in the pharmaceutical industry as a digestive aid [2]. Although, amylases have been used in various industries, the production of the enzyme has been limited to selected strains of fungi and bacteria. Moreover, the demand for this enzyme is further limited to specific applications if only fungi and bacteria are employed for the production of the enzyme [4]. Therefore, there is a need to find new sources for the production of the valuable enzyme. It is well documented that plants are abundant source of amylases [4], and using plants as an alternative source of the enzyme is promising, as plants offer advantages over traditional microbial sources, including low production cost, ease of scale-up, and availability of natural storage organs [5].

Dragon (*Hylocereus polyrhizus*) or dragon fruit has already received worldwide recognition as an ornamental plant due to its angular stems and primarily white, scented, night-blooming flowers [6]. The fruits of* Hylocereus polyrhizus*, known as red dragon, have recently drawn the close attention of growers worldwide because of their economic value and potential health benefits. These fruits are currently being grown commercially in Malaysia, Taiwan, Vietnam, South China, Nicaragua, Colombia, Australia, and the USA, and it is expected that the area occupied by dragon farming will increase substantially due to high demand [7]. Thus, it can be concluded that dragon is one of the most important commercial tropical fruits in the world. Approximately 33% of the whole fruit weight is in the peel [8], which is often discarded during processing, especially in the beverage production industry, as waste or used as animal feed. These discarded peels pose an environmental risk, and the subsequent treatment of such waste has been very costly for industry. However, there are different types of enzymes in the peel; thus, the peel can be used as a rich and cost-effective source for the commercial production of natural and valuable enzymes such as amylase.

The alteration or destruction of the natural morphology of an enzyme, which causes a decrease in enzyme activity and stability, is due to undesirable extraction conditions. Therefore, it is crucial to optimise the extraction process to produce enzymes with high activity and stability. The main objective of the present study was to investigate the effect of extraction conditions on enzymatic properties of amylase derived from dragon peel. It should be noted that the extracted amylase enzyme from dragon peel showed specific characteristics such as thermostability at high temperature, high activity at acidic pH, and high stability of the enzyme in presence of ionic and nonionic surfactant agents. The optimisation of the extraction process should result in achieving the maximum enzyme activity, specific activity, temperature stability, pH stability, surfactant agent stability, and storage stability. The amylase extraction conditions included the buffer-to-sample (B/S) ratio (2 : 1 to 6 : 1, w/w), mixing time (60 to 180 seconds), extraction temperature (−18 to 25°C), and the pH of the buffer (2.0 to 8.0). It should be noted that no study concerning the optimization of amylase extraction from dragon peel has been performed to date.

## 2. Methods and Materials

### 2.1. Chemicals and Plant Material

All chemicals and reagent were in analytical grade. Bradford reagent, bovine serum albumin (BSA), and 3,5-dinitrosalicylic acid (DNS) were obtained from Sigma Chemical Co., (St. Louis, MO, USA). Dibasic sodium phosphate (Na_2_HPO_4_ *·*2H_2_0), monobasic sodium phosphate (NaH_2_PO_4_ *·*H_2_0), sodium acetate, acetic acid, sodium citrate, citric acid, soluble starch, maltose, and sodium potassium tartrate (NaKC_4_H_4_O_6_ *·*4H_2_O) were obtained from Merck (Darmstadt, Germany). Red dragon fruits (*Hylocereus polyrhizus*) were purchased from Passer Brong (Selangor, Malaysia). Ripened dragon fruits were selected based on the size uniformity at the same stage of ripening and free of visual defects. The fruits were stored in a cold room at 4°C until used for extraction procedure.

### 2.2. Extraction of Amylase Enzyme from Dragon Peel

Fresh dragon fruits (2 kg) were cleaned and rinsed thoroughly with sterile distilled water. The peels of dragon were removed and chopped into small pieces (1 cm^2^ each, 1 mm thick); then, they were quickly blended (model 32BL80, Dynamic Corporation of America, New Hartford, CT, USA) with buffer under different enzyme extraction conditions (i.e., B/S ratio (2 : 1 to 6 : 1)), temperature (−25°C to +25°C), pH (2–8), and mixing extraction time (60 to 180 seconds). In this experiment, the pH was adjusted using 0.1 M glycine-HCl buffer (pH 2.0), sodium acetate buffer (pH 5.0) and Tris-HCl buffer (pH 8.0). The peel-buffer homogenate was filtered through cheesecloth. The filtrate was centrifuged at 6000 rpm for 5 min at 4°C, and the supernatant was then collected. The supernatant (crude enzyme) was stored at 4°C for future analysis.

### 2.3. Analytical Procedure

#### 2.3.1. Amylase Activity Assay and Protein Concentration Determination

The amylase activity was determined according to the method of Kammoun et al. [9] with slight modifications. The reaction mixture consisted of 500 *μ*L of crude enzyme and 500 *μ*L of 0.1% soluble starch prepared in 0.1 M sodium acetate buffer at pH 5.0. The reaction mixture was incubated at 70°C for 30 min. Subsequently, 1000 *μ*L of DNS was added to the reaction mixture and then heated in boiling water for 5 min; the mixture was then cooled in tap water. The released reducing sugar was determined by spectrophotometry (BioMate-3, Thermo Scientific, Alpha Numerix, Woodfield Dr, Webster, NY, USA) at 540 nm using maltose as a standard reducing sugar. One unit of *α*-amylase activity was defined as the amount of enzyme that produced 1 *μ*mol of maltose per minute under the enzyme activity conditions applied. The protein concentration was determined by the Bradford [10] method, using BSA as a standard.

#### 2.3.2. Determination of Specific Activity

To evaluate the extraction process of the enzyme, the given (1) was used to measure the specific activity [10]

(1) $$\begin{array}{c} \text{Specific}\;\;\text{activity}\left(\text{U}/\text{mg}\right)=\frac{\text{Total}\;\;\text{activity}\left(\text{U}\right)}{\text{Total}\;\;\text{Protein}\left(\text{mg}\right)}. \end{array}$$

#### 2.3.3. Determination of Surfactant Stability

The stability of amylase in the presence of certain surfactants (i.e., triton X-100, Tween 80, and SDS) was investigated. The test tube used contained 500 *μ*L of crude enzyme and 500 *μ*L (5 mM) of surfactant; the tube was preheated for 30 min at 25°C. Then, the residual activity of the enzyme was determined by performing an amylase activity assay as described above, and the results were compared to those obtained in control tubes incubated without surfactant [1]. Assays were performed in triplicate, and the resulting activity in the test tube was expressed as a percentage of that obtained in the control tubes.

#### 2.3.4. Determination of Temperature Stability

The thermostability of amylase was determined by incubating 500 *μ*L of the extracted enzyme in 500 *μ*L of 50 mM acetate buffer (pH 5.0) at 20°C, 30°C, 40°C, 50°C, 60°C, 70°C, and 80°C for 30 min. The residual enzymatic activity was then measured using an amylolitic enzyme activity assay [11].

#### 2.3.5. Determination of pH Stability

The effect of pH on the amylase stability was determined by incubating the reaction mixture at various pH levels ranging from 2 to 10 using the following buffers: 0.1 M glycine-HCl (pH 2.0–3.5), 0.1 M sodium acetate (pH 4.0–5.5); 0.1 M sodium phosphate (pH 6.0–7.5); 0.1 M Tris-HCl (pH 8.0–9.0); 0.1 M glycine-NaOH (pH 9.5–10). To test its pH stability, the extracted enzyme was preincubated at different pH levels in the mixed buffer for 30 min at room temperature prior to the assay [12]; the remaining enzyme activity was then determined using the standard assay as mentioned previously.

#### 2.3.6. Determination of Storage Stability

Extracted enzyme was stored for a week at 4°C. The amylase enzyme activity was evaluated after the storage following the standard assay condition. The ratio of amylase activity after storage time to the initial enzyme activity was given the efficiency of enzyme storage stability:

(2) $$\begin{array}{c} \text{Storage}\;\;\text{stability}\left(\%\right)=\frac{A}{A_{0}}\times 100, \end{array}$$

where *A* is enzyme activity of amylase after storage time and *A*_0_ is initial enzyme activity of amylase [13].

### 2.4. Experimental Design

Response surface methodology using central composite design (CCD) was employed to determine the effect of the enzyme extraction conditions, that is, B/S ratio (2 : 1 to 6 : 1), extraction temperature (−18° to +25°C), mixing time (60 to 180 seconds), and buffer pH (2.0 to 8.0), on the enzymatic properties of amylase from dragon peel. Thirty treatments were assigned based on CCD, with four independent variables at five levels for each variable, including six centre points, eighteen factorial points, and six star (axial) points. The enzymatic properties of amylase, such as enzyme activity, specific activity, thermostability, pH stability, storage stability, and stability in the presence of surfactants, were considered as response variables. Experiments were randomised to minimise the effects of unexplained variability in the actual responses due to extraneous factors [14].

### 2.5. Statistical Analysis and Optimization Procedure

Response surface analysis was performed to determine the regression coefficients and statistical significance of the model as well as fit the regression models to the experimental data to achieve an overall optimum region for all response variables studied. The optimum amylase extraction condition was predicted according to the following equation:

(3) Y=β0+β1X1+β2X2+β3X3+β4X4+β11X12 +β22X22+β33X32+β44X42+β12X1X2 +β13X1X3+β14X1X3+β23X2X3+β24X2X4.

The graphical optimization procedure was expressed as three-dimensional (3D) response surface plots. Overlaid plots were drawn to visualise the significant (*P* < 0.05) interaction effects of the enzyme extraction variables on the enzymatic properties of the amylase extracted from dragon peel. In addition, numerical optimization using a response optimizer was applied to determine the exact optimum points of the independent variables that enhance the optimum enzyme extraction procedure such that amylase exhibits desirable enzymatic properties. The experimental data were compared with the fitted values predicted by the response regression equations to verify the accuracy of the final reduced models. The Minitab version 16 (Minitab Inc., State College, PA, USA) software package was used to design and analyse the experimental data.

## 3. Result and Discussion

### 3.1. Fitting the Initial Response-Surface Models

In the present work, multiple regression analysis was performed using response surface analysis to develop a relationship between four enzyme extraction variables and the enzymatic properties of amylase extracted from dragon peel, as presented in Table 1. It should be noted that the nonsignificant (*P* < 0.05) terms were dropped from the initial model, and the experimental data were then refitted simply using the significant (*P* < 0.05) terms. The nonsignificant (*P* > 0.05) terms were retained in the final reduced model if the quadratic or interaction effect significantly affected the response variables. As indicated by (4), the model obtained yielded the main, quadratic, and interaction effects of factors affecting the response variables. The estimated regression coefficients of the main, quadratic, and interaction effects deemed significant and their corresponding *R* ^2^ values are shown in Table 1; an indication of lack of fit is also presented. The significance of each term was determined using the *F*-ratio and *P* value, as presented in Table 2. The results also indicate that the regression models for all response variables were significant according to the *F*-test at the 5% confidence level (*P* < 0.05). In addition, the *P* values of all of the regression models were less than 0.005 (Table 1), which confirmed that there was no lack of fit. The *R* ^2^ values for amylase activity, specific activity, surfactant agent, thermal stability, pH stability, and storage stability were 0.983, 0.968, 0.913, 0.894, 0.900, and 0.935, respectively. Thus, the *R* ^2^ values for all response variables were higher than 80% (89.4%–98.3%), and the response surface models were suitably and accurately used for predicting a high percentage of variation (80%) in the properties of extracted amylase as a function of the extraction variables. The lack of fit, which indicates the fitness of models, showed that there was no significant *P* value (*P* > 0.05) in terms of the response variables studied at the 95% confidence level, which confirmed the sufficient fitness of the regression model with experimental values. Among all of the enzyme extraction variables, the B/S ratio and temperature showed the most and least significant (*P* < 0.05) effects on the enzymatic properties of amylase from dragon peel, respectively (Table 2). The results also indicate that the extraction variables were primarily affected by the interaction of the mixing time and B/S ratio. The response surface models, described by (4), were fitted to each of the response variables (*Y*) and four independent variables (*X*_1_, *X*_2_, *X*_3_, and *X*_4_).

Formula is as follows:

(4) Y1=64.83+48.16X1+64.65X2+95.10X3+139.56X12+134.49X22+138.92X32+101.78X42+68.21X1X2+45.09X2X4+68.02X3X4,Y2=14.18+1.27X1+2.54X2+2.76X3+5.11X4+2.77X12+1.68X22+2.20X32+2.42X42+1.94X2X3+2.91X2X4,Y3=89.91+7.28X2+13.79X3+10.83X4+12.64X22+10.64X32+15.14X42+8.59X2X4,Y4=83.52+5.43X1+8.14X2+9.72X3+14.40X4+6.78X12+11.31X22+11.08X32+8.53X42+9.31X2X3+8.18X3X4,Y5=87.76+3.43X1+9.16X2+9.03X3+14.53X4+8.33X12+13.59X22+13.47X32+8.72X42+13.80X2X3+9.37X3X4,Y6=82.56+11.70X2+7.66X3+10.28X4+8.27X22+8.91X32+11.93X42+5.80X2X4+13.47X3X4.

### 3.2. Activity of Amylase

**Figure 1:**
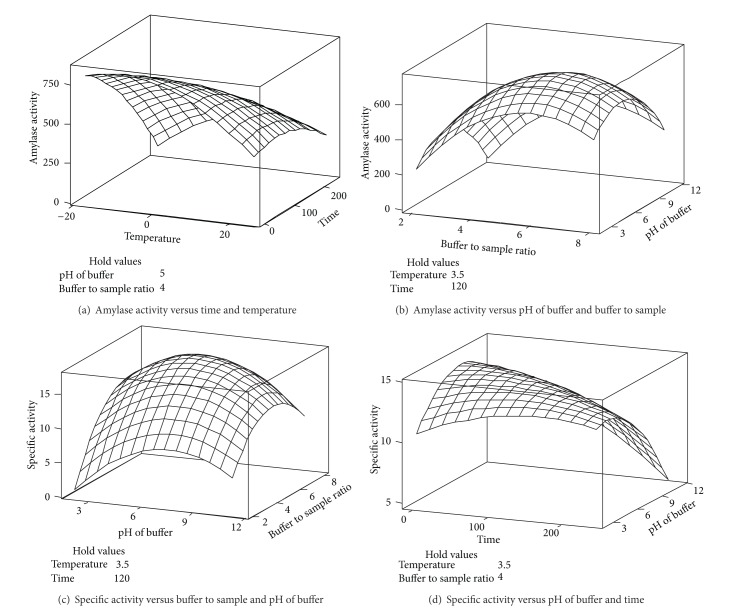
Three-dimensional curves for showing the significant interaction effect of independent extraction variables (e.g., temperature, time of mixing, pH of buffer, and buffer to sample ratio) on activity (a-b) and specific activity (c-d) of amylase were plotted.

As shown in Table 2, all of the main and quadratic effects of the extraction variables as well as the interaction effects of temperature with time, time with B/S ratio, and buffer pH with the B/S ratio were significantly (*P* < 0.05) fitted to the amylase activity model. Conversely, the interaction effect of time with the buffer pH, temperature with the B/S ratio, and time with the B/S ratio indicated insignificant (*P* < 0.05) effects on the enzyme activity (Table 2). Based on the *F*-ratio, the most significant extraction variables with respect to amylase activity were B/S ratio, pH of buffer, mixing time, and temperature (Table 2). It should be noted that the main effect of the B/S ratio showed the most significant (*P* < 0.05) effect on the activity of amylase because it exhibited the highest *F-*ratio; in fact, increasing the B/S ratio caused an increase in the amylase activity because of the greater binding capacity of the buffer toward the active site of the enzyme during the extraction procedure. Rocha and Morais [15] also reported that an increase in the B/S ratio caused an increase in the activity of polyphenoloxidase extracted from “Jonagored” apple. It should be noted that the enzyme activity decreased with decreasing amounts of buffer due to the difficulty of homogenising the sample with buffer; a decrease in the enzyme solubilisation of the crude extract was also observed [15, 16]. Temperature and time are two important physical parameters in enzyme extraction, and it was observed that the main term of temperature had the least significant effect on the enzyme activity. As shown in Figure 1(a), the most suitable temperature for the extraction of amylase was determined to be 0°C, but the activity of the enzyme was not significantly altered at lower or higher temperatures (−18°C and 25°C). This behaviour could be due to the thermal stability of the enzyme at the temperatures considered in this study. Long mixing time showed a negative effect on the enzyme activity. This effect could imply that increasing the extraction time might increase the probability of the enzyme contacting the buffer, but the negative effect of higher shear forces during the mixing procedure could denature and deactivate the enzyme. Thus, the enzyme activity was significantly (*P* < 0.05) reduced at longer mixing times during amylase extraction (Figure 1(b)). As shown in (Figure 1(b)), the interaction effect of the buffer pH and B/S ratio showed a significant (*P* < 0.05) effect on the activity of amylase. It should be noted that the pH value of a liquid medium affects the structure of an enzyme. In fact, enzymes have ionic groups on their active sites, which must remain in their stable form. The variation in the pH of the medium results in changes in the ionic form of the active site, which affects the reaction rate [17]. As shown in Figure 1(b), the highest enzyme activity (648.4 U) was obtained at pH 5.0. It should be noted that the activity of amylase from* Anoxybacillus flavithermus* [18],* Bacillus *sp. YX-1 [19],* Nesterenkonia *sp. [20], and* Eisenia foetida* [21] was 438.6 U, 530 U, 262 U, and 549 U, respectively. The result indicates that the enzyme at this pH was in its stable form and the highest reaction rate between the active site of the enzyme and the substrate was achieved at this pH.

### 3.3. Specific Activity

Enzyme activity and protein concentration are two important parameters that determine the specific activity of amylase. Thus, all variables such as time, B/S ratio, buffer pH, and temperature that affect the activity of amylase could also have an effect on the specific activity of the enzyme. Differences in protein concentration create differences between the specific activity and activity of an enzyme. The result shows that the main effect of mixing time and the B/S ratio showed the most significant (*P* < 0.05) effect on the specific activity of amylase (Table 2). In addition, the interaction effect of mixing time and B/S ratio was one of the most significant (*P* < 0.05) interactions affecting the specific activity (Table 2). This finding indicates that the mixing time affected the total protein content of the extracted sample; in fact, increasing the mixing time above the optimum point caused the protein/enzyme to denature due to the loss of tertiary structure. In addition, it was observed in our study that increasing the mixing time led to the emergence of unwanted proteins or contaminants in the extracted sample. It was observed that the specific activity was increased by increasing the B/S ratio from 2 : 1 to 4 : 1 (Figure 1(c)), whereas an increase in the B/S ratio to 6 : 1 caused a decrease in the specific activity of amylase, which could be due to the inordinately high dilution of the enzyme extraction solution. A similar observation was made by Mukerjea et al. [22], who investigated the effect of buffer dilution on the activities of potato starch synthesis and starch branching enzymes. The researchers also observed that increasing the concentration of the buffer to a certain point resulted in an increase in enzyme activity, but dilution of the enzyme beyond that point resulted in zero enzyme activity.

According to the results obtained in this study (Table 2), the interaction effect of the mixing time and pH of the buffer also showed a significant (*P* < 0.05) effect on specific activity. As shown in Figure 1(d), the specific activity of the enzyme was decreased at alkaline pH, and the highest specific activity was obtained at pH 5.0, which confirmed that the enzyme is stable at acidic pH. On the other hand, the main and quadratic terms of temperature showed the least significant (*P* < 0.05) effects on the specific activity of amylase, similar to the effect of this independent variable on the enzyme activity (Table 2), which could be due to the stability of the enzyme at temperatures used for extraction in this study. Thus, the minimum protein concentration (45.66 mg) that is desirable for achieving the highest specific activity (14.2 U/mg) was obtained at a B/S ratio of 1 : 4, mixing time of 120 seconds, temperature of 3.5°C, and pH 5.0.

### 3.4. Temperature Stability

As proteins, enzymes exhibit a three-dimensional structure. Small changes in the active sites of enzymes can result in a loss of stability. Temperature is one of the important parameters that can cause enzyme denaturation by enhancing molecular vibration, which is responsible for the breaking of intramolecular bonds [22]. Based on the results shown in Table 2, the main, quadratic, and interaction effects of temperature did not show any significant (*P* < 0.05) effect on the temperature stability of amylase. In fact, the enzyme retained approximately 88.4% stability at different extraction temperatures. It can be concluded that the enzyme was stable at different temperatures, from freezing to room temperature. This finding is in agreement with the results of Nerkar et al. [24], who reported that *α*-amylase from* mat* bean (*Phaseolus aconitifolius*) is thermostable. It should be considered that the temperature stability of amylase is one of the most attractive characteristics of the enzyme. The advantages of thermostable amylases, especially in industrial processes, are a reduced risk of contamination, low external cooling costs, high substrate solubility, and low viscosity, allowing for accelerated mixing.

**Figure 2:**
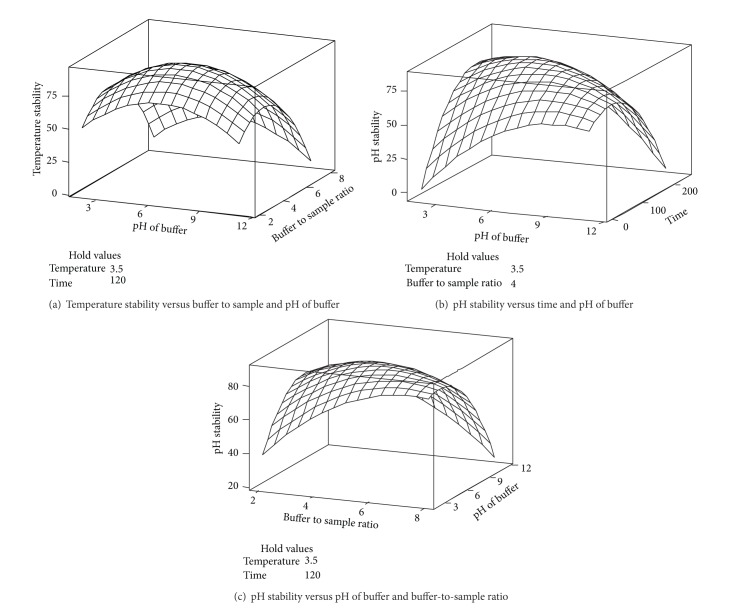
Three-dimensional surface plots for showing the significant interaction effect of independent extraction variables on temperature stability (a) and pH stability (b-c) of amylase were plotted.

It should be noted that temperature stability was positively proportional to the main effects of time, pH, and the B/S ratio (Table 1). In addition, among all of the interaction effects, the interaction effect of time and the B/S ratio had a significant (*P* < 0.05) effect on temperature stability. A 3D surface plot was graphed to visualise the significant (*P* < 0.05) interaction effect of the enzymatic extraction variables on temperature stability (Figure 2(a)). As shown in Figure 2(a), the temperature stability of the enzyme at different extraction temperatures was increased by increasing the time and B/S ratio. In fact, the temperature stability of the enzyme was increased by simultaneously increasing the B/S ratio and time up to a certain level. Thus, the high levels of the interaction variables, above their optimum points, observed during the extraction procedure caused a decrease in the temperature stability of amylase. On the other hand, temperature did not show any significant effect, thus confirming the stability of enzyme temperature (Table 2).

### 3.5. pH Stability

As shown in Table 2, the main effects of all of the independent variables indicated significant (*P* < 0.05) effects on the pH stability, but the main term of pH had the most significant (*P* < 0.05) effect on the pH stability of amylase among the other extraction variables (*F*-ratio: 24.90, Table 2). The results indicate that the enzyme retained 85.2% of its original activity at pH 5.0, and the highest enzyme activity was achieved at this pH (Figure 2(b)). The observation of the maximum activity of amylase at low pH values and the good stability of the enzyme are very important from an applications perspective. Most amylase enzymes are unstable at low pH [24, 25], exhibiting the highest enzyme stability at pH values of 5.0–8.0. In addition, there are several industrial processes that are performed at low pH and thus require enzymes with low-pH stability. For example, amylase extracted and purified from Korean pine seeds by Azad et al. [13] showed maximum enzyme stability at pH 5.0. Noman et al. [26] also stated that the optimum pH for amylase extracted from the tuber* Pachyrhizus erosus* L. was achieved at pH 7.0. Therefore, the fact that amylase exhibits optimum activity at low pH values makes the enzyme highly attractive for application in various industrial procedures. It should be noted that enzymes possess maximal activity at their isoelectric points due to the minimisation of electrostatic repulsion forces resulting from the maximum interaction with the surrounding buffer and the increase in enzyme activity. The isoelectric point of amylase is 4.7. Thus, the highest activity of amylase was obtained at pH 5.0, close to the isoelectric point of the enzyme (Figure 2(b)).

Based on the results, the interaction effect of the buffer pH and time and the interaction effect of the buffer pH and B/S ratio indicated significant (*P* < 0.05) effects on the pH stability of amylase (Table 2). As shown in Figure 2(b), the pH stability of amylase was decreased at pH values of 2.0 and 8.0. This phenomenon could imply that amylase will be denatured and will unfold, causing a loss of both structure and stability, at very low (acidic) and high (alkaline) pH values. In addition, it should be noted that structural perturbations under extreme pH conditions occurred due to the disruption of electrostatic interactions, which play an important role in protein stability. Moreover, the presence of curvature in Figure 2(c) indicates that the interaction effect of the buffer pH and B/S ratio significantly (*P* < 0.05) affected the pH stability in a parabolic manner. This finding suggests that the maximum stability of the enzyme structure was achieved by increasing the pH of the buffer to 5.0 and increasing the B/S ratio from 1 : 2 to 1 : 4 (Figure 2(c)).

### 3.6. Surfactant Agent Stability

**Figure 3:**
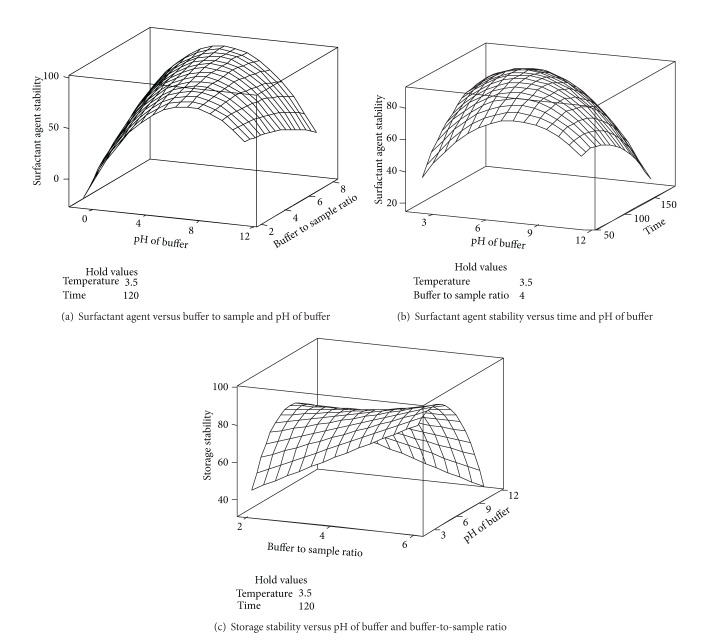
Three-dimensional surface plots for showing the significant interaction effect of independent extraction variables on surfactant agent stability (a-b) and storage stability (c) of amylase were plotted.

The surfactant agent stability of an enzyme is one of the important parameters that enable an enzyme to be used in different industries, such as the detergent industry. Thus, to achieve the highest surfactant agent stability of the enzyme is one of the main goals for the optimization of the amylase extraction procedure. Most surfactants that interact with proteins create distinct electrostatic and hydrophobic regions and alter the secondary or tertiary structure of enzymes [27]. In addition, it has been reported that it is the enzyme substrate that interacts with surfactants instead of the enzyme itself. Thus, the hydrolysis of an enzyme would be affected by changes in the molecular structure of the enzyme and substrate. Therefore, some enzymes are unstable and unfolded by surfactant agents, whereas amylase from dragon peel was observed to retain 87.2% of its activity in the presence of surfactants used at a concentration of 10% (w/v). A similar observation was reported by Lin et al. [1], who partially purified amylase extracted from soybean. The authors explained that the surfactant agent stability of the enzyme might be due to the fact that, in starch, amylase and—to a lesser extent—amylopectin can interact with surfactants to give rise to an inclusion complex. The formation of these complexes between the surfactant and starch can hamper the enzymatic hydrolysis of amylase [28]. Based on the results obtained in this study (Table 1), the final reduced model fit the surfactant agent stability and showed a relatively high *R* ^2^ with no indication of significant (*P* > 0.05) lack of fit. This finding indicates a satisfactory fitness of the surfactant agent stability model as a function of the enzymatic extraction variables (Table 1). The main and quadratic effects of all of the extraction variables significantly (*P* < 0.05) affected the surfactant agent stability (Table 2). It should be noted that the sole effect of the B/S ratio was negatively related to the surfactant agent stability of amylase, but the interaction effect of the B/S ratio with time was positively proportional to the response (Table 2). This result confirms that the B/S ratio had both positive and negative effects on the surfactant agent stability. In fact, increasing the B/S ratio up to a certain point increased the stability of amylase, but further increasing of the B/S ratio led to a decrease in stability (Figure 3(a)).  Figure 3(b) shows the interaction effect of mixing time and pH of buffer on surfactant agent stability of extracted amylase. This figure indicates that increasing of time and pH of buffer to certain level (i.e., 2 min mixing time; 5.0 pH of buffer) caused increment of surfactant agent stability. In contrast, the stability of the enzyme was reduced at longer times and at alkaline pH. This inhibitory effect on the surfactant agent stability of amylase could be due to the combined effect of various factors, such as the reduction in hydrophobic interactions, which play a crucial role in stabilising the tertiary structure of proteins.

### 3.7. Storage Stability

Storage stability is one of the most important parameters to be considered in enzyme extraction procedures. Table 1 shows that the main effect of the B/S ratio and the interaction effect between mixing time and buffer pH were positively related to the storage stability of amylase. In contrast, temperature did not show any significant effect on the storage stability of the enzyme after one week, a favourable characteristic exhibited by the enzyme in this study. In addition, the sample extracted at pH 5.0 demonstrated the highest storage stability, which suggests that the active site of the enzyme is stable at this pH and could properly interact with the substrate after the storage time. The enzyme extracted at 2 min was more stable than the other samples submitted to shorter or longer mixing times, which indicates that the tertiary structure of the enzyme was not damaged during this mixing period. A 3D surface plot was drawn to visualise the significant (*P* < 0.05) interaction effects of the extraction variables on amylase.  Figure 3(c) shows the interaction effect of pH of buffer and buffer-to-sample ratio on storage stability of extracted amylase. Therefore, the figure explores that increment of pH of buffer up to pH 5.0 in terms of increasing of buffer-to-sample ratio until 6 : 1 induced to enhancement of storage stability of the amylase. In fact, performing the extraction using six times as much acidic buffer led to an increase in the storage stability of amylase (Figure 3(c)). This result indicates that the buffer concentration altered the microenvironment of the enzyme by increasing the compactness of the protein structure [29]. Asada et al. [30] also reported that using a highly concentrated buffer increases both the thermodynamic and kinetic stability of an enzyme. The authors further mentioned that the compactness of horseradish peroxidase was increased at a high concentration of buffer. Thus, as can be clearly observed in Figure 3(c), the enzyme storage stability was significantly reduced when the B/S ratio was increased from 4 : 1 to 6 : 1. The highest stability of amylase observed after one week of storage at 3.5°C was 90.3%.

### 3.8. Optimization and Validation Procedures

In this study, the most suitable conditions for amylase extraction were considered to be those that resulted in the highest enzyme activity, enzyme specific activity, temperature stability, pH stability, surfactant agent stability, and storage stability. Overall, the optimum extraction conditions were obtained by graphical and numerical optimization. Multiple graphical optimizations were carried out by overlaying counter plots to determine the optimum region of amylase extraction conditions. Therefore, the selected extraction conditions led to the extraction of amylase with desirable enzymatic properties from dragon peel.

**Figure 4:**
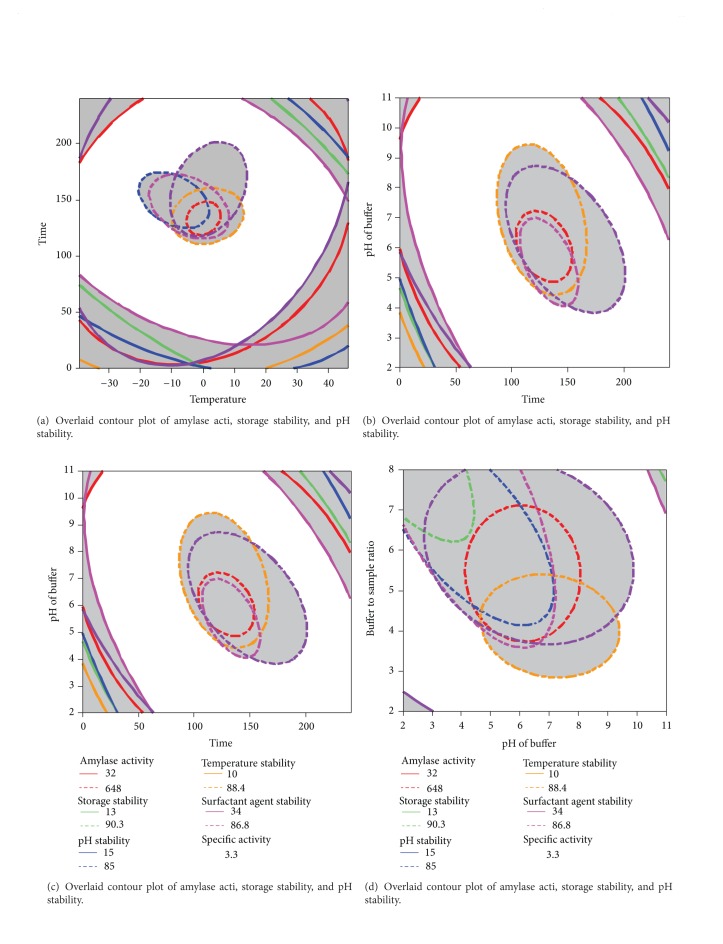
Graphical optimization using overlaid counter plots for showing the optimum area of amylase extraction variables was plotted.

For the graphical optimization process, 3D response surface plotting was used, followed by the superposition of all 3D plots to determine the optimum conditions [14].  Figure 4 indicates the significant (*P* < 0.05) interaction effects of four of the enzyme extraction variables on the enzymatic properties of amylase from dragon peel. In addition, numerical optimization was performed to determine the exact optimum level of the extraction variables. The numerical optimization demonstrated that the most desirable conditions for amylase extraction from dragon peel were achieved at a B/S ratio of 1 : 4 (w/w), mixing time of 120 seconds, temperature of 3.5°C, and buffer pH of 5.0. Under the optimum conditions recommended by the response surface analyser, the following values were predicted for the extraction of the enzyme based on the final reduced models: amylase activity 648.4; specific activity, 14.2 U/mg; temperature stability, 88.4%; pH stability, 85.2%; surfactant agent stability, 86.8%; and storage stability, 90.3%. Thus, the enzyme was extracted under the recommended optimum extraction conditions. The validity of the optimum conditions was determined by comparing the enzymatic properties of the amylase experimentally produced under the optimum conditions and the data predicted from the models. Under the optimum extraction conditions, the following enzymatic properties were experimentally obtained: amylase activity, 623.2; specific activity, 13.9 U/mg; temperature stability, 82.1%; pH stability, 80.4%; surfactant agent stability, 87.2%; and storage stability, 87.7%. No significant (*P* > 0.05) difference was observed between the enzymatic properties of the extracted amylase and the predicted values achieved from the final reduced models. Thus, the similarity between the experimental and predicted enzymatic characteristics confirmed the adequacy of the corresponding models in predicting the variation in the extracted amylase properties.

## 4. Conclusion

In the present study, the enzymatic properties of amylase were shown to be significantly (*P* < 0.05) affected by the main extraction variables considered. Response surface analysis led to significant (*P* < 0.05) regression models that showed no indication of lack of fit, thus ensuring reliable adjustments between the independent and response variables. The results demonstrated that the activity and stability of amylase extracted from red dragon peel were significantly (*P* < 0.05) affected by changes in the B/S ratio as well as mixing time. The fact that temperature showed the least significant effect (*P* < 0.05) on enzymatic activity and no significant (*P* < 0.05) effects of this variable on temperature stability were observed confirmed that the enzyme is thermostable. Because enzymes are heat sensitive, with high temperatures causing denaturation, the thermostability of the enzyme extracted from dragon peel is one of the important parameters to consider in the application of amylase in different industries. The enzyme also showed the highest enzyme activity and stability at pH 5.0, whereas the stability of the enzyme was significantly (*P* < 0.05) reduced at pH 8.0, which could be due to denaturation of the enzyme at alkaline pH. The extraction of the enzyme under controlled conditions demonstrated the high stability of the amylase in the presence of surfactant agents, which is also a favourable enzyme property. Thus, based on the results obtained in the study, the B/S ratio and the mixing time should be considered the most important parameters for the extraction of amylase from dragon peel. It was observed that the enzyme was best extracted from dragon peel using a B/S ratio of 1 : 4, mixing time of 120 seconds, temperature of 3.5°C, and pH 5.0. The results of this study show that the natural and valuable enzyme amylase, which exhibits unique characteristics such as thermostability, high stability at low pH, and surfactant agent stability, can be used as a potential low-cost enzyme in different industries and biotechnological applications.

## Figures and Tables

**Table 1:** Regression coefficients, *R* ^2^, and *P* value of lack of fit for the final reduced models.

Regression coefficient	Amylase activity (*Y*_1_, U/mL)	Specific activity (*Y*_2_, U)	Temperature stability (*Y*_3_, %)	pH stability (*Y*_4_, %)	Surfactant stability (*Y*_5_, %)	Storage stability (*Y*_6_, %)
*b*_0_	64.83	14.18	89.91	83.52	87.76	82.56
*b*_1_	48.16	1.27	2.36	5.43	3.43	0.99
*b*_2_	64.65	2.54	—	8.14	9.16	—
*b*_3_	95.10	2.76	13.79	9.72	9.03	7.66
*b*_4_	12.34	5.11	10.83	14.40	−14.53	10.28
*b*_1_^2^	139.56	2.77	—	6.78	8.33	11.42
*b*_2_^2^	134.49	1.86	12.64	11.31	13.59	8.27
*b*_3_^2^	138.92	2.20	10.64	11.08	13.47	8.91
*b*_4_^2^	101.78	2.42	15.14	8.53	8.72	11.93
*b*_1_ *b*_2_	68.21	—	—	—	—	—
*b*_1_ *b*_3_	—	—	—	—	—	—
*b*_1_ *b*_4_	—	—	—	—	—	—
*b*_2_ *b*_3_	—	1.94	—	9.31	13.80	8.76
*b*_2_ *b*_4_	45.09	—	8.59	—	—	—
*b*_3_ *b*_4_	68.02	0.85	—	8.18	9.37	13.47
*R* ^2^	0.983	0.968	0.913	0.894	0.900	0.935
*P* value	0.000∗	0.002∗	0.003∗	0.005∗	0.001∗	0.001∗
Lack of fit (*P* value)	205.77	395.10	202.99	130.89	160.02	120.01

^1^Temperature; ^2^time of mixing; ^3^pH of buffer; ^4^buffre: sample (B/S) ratio.

∗Significant (*P* < 0.05); *b*_*i*_, *b*_*ii*_ and *b*_*ij*_: the estimated regression coefficient for the main linear quadratic and interaction effects, respectively.

**Table 2:** *F*-ratio and *P* value for each independent variable effect in the polynomial response surface models.

Variables		Main effects				Quadratic effects				Interaction effects					
		*X*_1_	*X*_2_	*X*_3_	*X*_4_	*X*_1_^2^	*X*_2_^2^	*X*_3_^2^	*X*_4_^2^	*X*_1_ *X*_2_	*X*_1_ *X*_3_	*X*_1_ *X*_4_	*X*_2_ *X*_3_	*X*_2_ *X*_4_	*X*_3_ *X*_4_
Amylase activity (*Y*_1_, U/mL)	*P* value	0.033∗	0.002∗	0.000∗	0.000∗	0.045∗	0.000∗	0.001∗	0.000∗	0.023∗	—	—	—	0.015∗	0.011∗
	*F*-ratio	7.84	19.20	39.69	86.06	5.76	14.44	17.64	50.41	10.17	—	—	—	12.69	13.96
Specific activity (*Y*_2_, U)	*P* value	0.040∗	0.002∗	0.013∗	0.001∗	0.033∗	0.001∗	0.006∗	0.004∗	—	—	—	0.001∗	0.010∗	—
	*F*-ratio	6.81	27.14	12.39	42.40	8.00	33.40	12.58	19.09	—	—	—	39.94	13.54	—
Temperature stability (*Y*_3_, %)	*P* value	—	0.000∗	0.014∗	0.000∗	—	0.001∗	0.008∗	0.000∗	—	—	—	—	0.017∗	—
	*F*-ratio	—	28.70	8.09	28.17	—	19.89	9.78	20.16	—	—	—	—	7.44	—
pH stability (*Y*_4_, %)	*P* value	0.043∗	0.005∗	0.000∗	0.015∗	0.021∗	0.001∗	0.001∗	0.006∗	—	—	—	0.025∗	—	0.044∗
	*F*-ratio	3.34	11.42	24.90	7.95	7.09	12.60	18.92	5.29	—	—	—	5.05	—	1.78
Surfactant stability (*Y*_5_, %)	*P* value	0.040∗	0.013∗	0.015∗	0.001∗	0.011∗	0.008∗	0.001∗	0.000∗	—	—	—	0.004∗	—	0.035∗
	*F*-ratio	1.12	8.58	8.35	21.52	9.12	4.84	11.02	24.30	—	—	—	12.18	—	10.98
Storage stability (*Y*_6_, %)	*P* value	—	0.003∗	0.036∗	0.001∗	—	0.030∗	0.034∗	0.014∗	—	—	—	0.021∗	—	0.000∗
	*F*-ratio	—	17.05	5.76	21.06	—	6.65	6.22	15.52	—	—	—	7.84	—	37.94

*X*_1_, *X*_2_, *X*_3_, and *X*_4_: the main effect of temperature, time of mixing, pH of buffer and buffer to sample ratio, respectively. *X*_1_^2^, *X*_2_^2^, *X*_3_^2^, and *X*_4_^2^: the quadratic effect of effect of temperature, time of mixing, pH of buffer and buffer to sample ratio, respectively. *X*_1_ *X*_2_: the interaction effect of temperature and time of mixing; *X*_1_ *X*_3_: the interaction effect of temperature and pH of buffer, *X*_1_ *X*_4_: the interaction effect of temperature and buffer to sample ratio; *X*_2_ *X*_3_: interaction effect of time of mixing and pH of buffer; *X*_3_ *X*_4_: interaction effect of pH of buffer and Buffer to sample ratio.

∗Significant (*P* < 0.05).
